# Silent gene clusters encode magnetic organelle biosynthesis in a non-magnetotactic phototrophic bacterium

**DOI:** 10.1038/s41396-022-01348-y

**Published:** 2022-12-14

**Authors:** M. V. Dziuba, A. Paulus, L. Schramm, R. P. Awal, M. Pósfai, C. L. Monteil, S. Fouteau, R. Uebe, D. Schüler

**Affiliations:** 1 Department of Microbiology, Faculty of Biology, Chemistry and Geosciences, University of Bayreuth, Bayreuth, Germany; 2 Department of Microbial Biochemistry, Faculty of Life Sciences: Food, Nutrition and Health, University of Bayreuth, Bayreuth, Germany; 3 ELKH-PE Environmental Mineralogy Research Group, Veszprém, Hungary; 4 Research Institute of Biomolecular and Chemical Engineering, University of Pannonia, Veszprém, Hungary; 5 Aix-Marseille University, CEA, CNRS, Biosciences and Biotechnologies Institute of Aix-Marseille, Saint Paul lez Durance, France; 6 LABGeM, Genomique Metabolique, CEA, Genoscope, Institut Francois Jacob, CNRS, Universite d’Evry, Universite Paris- Saclay, Evry, France

**Keywords:** Bacterial genetics, Bacterial evolution, Microbial ecology

## Abstract

Horizontal gene transfer is a powerful source of innovations in prokaryotes that can affect almost any cellular system, including microbial organelles. The formation of magnetosomes, one of the most sophisticated microbial mineral-containing organelles synthesized by magnetotactic bacteria for magnetic navigation in the environment, was also shown to be a horizontally transferrable trait. However, the mechanisms determining the fate of such genes in new hosts are not well understood, since non-adaptive gene acquisitions are typically rapidly lost and become unavailable for observation. This likely explains why gene clusters encoding magnetosome biosynthesis have never been observed in non-magnetotactic bacteria. Here, we report the first discovery of a horizontally inherited dormant gene clusters encoding biosynthesis of magnetosomes in a non-magnetotactic phototrophic bacterium *Rhodovastum atsumiense*. We show that these clusters were inactivated through transcriptional silencing and antisense RNA regulation, but retain functionality, as several genes were able to complement the orthologous deletions in a remotely related magnetotactic bacterium. The laboratory transfer of foreign magnetosome genes to *R. atsumiense* was found to endow the strain with magnetosome biosynthesis, but strong negative selection led to rapid loss of this trait upon subcultivation, highlighting the trait instability in this organism. Our results provide insight into the horizontal dissemination of gene clusters encoding complex prokaryotic organelles and illuminate the potential mechanisms of their genomic preservation in a dormant state.

## Introduction

The discovery of various prokaryotic organelles during the last decades has led to the rejection of the previously common view that bacterial cells have simplistic organization [1]. One of the best-studied examples of such organelles are magnetosomes synthesized by magnetotactic bacteria (MTB). Magnetosomes consist of magnetic iron oxide or sulfide cores enveloped by a lipid bilayer and are aligned in one or multiple chains within the cells [2]. They enable MTB to passively align with the Earth’s magnetic field lines, which in combination with active cellular movement and a highly complex signal aerotactic transduction network facilitates their search for optimal redox conditions (magnetoaerotaxis) at the oxic-anoxic transition zones of the stratified aquatic habitats, which MTB populate ubiquitously and abundantly [3, 4]. The ability to exert precise biological control over the synthesis of these highly ordered mineral-containing structures has placed MTB in the spotlight of a growing number of studies focused on the genetics, molecular mechanisms, evolution, ecology, and biotechnological application of bacterial magnetic biomineralization [2, 5–7].

Formation of a membranous compartment, magnetic crystal synthesis, and assembly of magnetosome chains require the coordinated action of >30 magnetosome-associated proteins, which are encoded within several magnetosome gene clusters (MGCs). The evolutionary history of MGCs still represents a conundrum. Intriguingly, the ability to form magnetosomes is widely scattered on the bacterial tree of life: MTB are affiliated with *Alpha*-, *Delta*-, *Gamma*-, “*Ca*. Lambda-”, *Zeta*-, and “*Ca*. Etaproteobacteria” classes of phylum *Pseudomonadota*, as well as in the phyla *Nitrospirota*, *Nitrospinota*, “*Ca*. Omnitrophica”, “*Ca*. Latescibacteria”, *Planctomycetota, Fibrobacterota*, and “*Ca*. Riflebacteria” [8, 9]. Based on the broad phylogenetic distribution of MTB and the diversity of magnetosome crystal composition and morphology, multiple evolutionary origins of magnetotaxis had initially been proposed [10]. However, recent data indicate that at least the core set of magnetosome genes emerged only once and is highly conserved among all MTB [8, 11, 12]. Nevertheless, as to when they emerged and what was the further course of their evolution, is still a matter of vigorous debates [13, 14]. Phylogenetic trees built from 16 S rRNA or phylogenetic marker proteins are largely congruent with those based on the shared magnetosome proteins, suggesting potential vertical inheritance from a common ancestor before the emergence of the major phyla [12]. However, this scenario places the origin of this highly complex trait very close to the last common bacterial ancestor (LBCA), questioning the view on ancient prokaryotes as organisms with the primitive cellular organization. Additionally, this would imply numerous losses of magnetotaxis genes during phylogenetic divergence, which is not a parsimonious explanation, and give only marginal importance to the role that horizontal gene transfer (HGT) could play in the MGCs evolution [13, 14]. At the same time, multiple violations of the phylogenetic tree congruency were observed, indicating instances of HGT [9, 15–19]. Moreover, the potential ability for MGCs mobilization and transfer is supported by the fact that in many of the described MTB, MGCs are found in genomic regions of plasticity, so-called genomic magnetosome islands (MAI), which is supported by the deviating G + C content, codon adaptation index (CAI), tetranucleotide frequency and abundance of mobile elements [20, 21]. Furthermore, the ability of some hitherto non-magnetotactic organisms to biomineralize magnetosomes after receiving MGCs was recently demonstrated applying synthetic biology methods. Thus, the laboratory transfer of the major MGCs from a model MTB *Magnetospirillum gryphiswaldense* MSR-1 to a purple photosynthetic alphaproteobacterium *Rhodospirillum rubrum* and a closely related but non-magnetotactic *Magnetospirillum* sp. indeed endowed both species with magnetosome biosynthesis [22, 23]. These experiments indicated the sufficiency of the transferred gene set for magnetosome formation and the overall ability of these foreign hosts to integrate the magnetosome biosynthesis pathway into their metabolic networks, suggesting that this might also occur under natural conditions.

To which extent HGT impacts the evolution of magnetosome formation is not yet clear. It is generally assumed that the horizontally transferred genes remain fixed in the population if they are properly expressed and confer advantages to the host, i.e., improve their fitness [24]. Hence, the currently documented MTB diversity supposedly represents the product of natural selection for fixation of magnetosome formation as a favorable trait, whereas all the non-functional or non-beneficial gene acquisitions have been eliminated from the gene pool and therefore have remained unavailable for analysis or laboratory experiments to date. This “survival bias” severely limits our understanding of the fate of horizontally transferrable genes, especially for complex traits controlled by multiple genes.

Here, we report the discovery of the silent MGCs in the culturable non-magnetotactic phototrophic bacterium *Rhodovastum atsumiense* G2-11, which were apparently acquired by a recent HGT from an alphaproteobacterial MTB. We further present a comprehensive study of the transcriptional pattern, functionality, and fitness effect of the magnetosome genes in G2-11. Moreover, we “magnetized” G2-11 through artificial HGT of the major MGCs from MSR-1 under laboratory conditions and explored the trait stability upon subcultivations. Our results provide the first evidence for horizontal dissemination of gene clusters encoding bacterial magnetic organelles outside MTB and illuminate the potential mechanisms of their preservation in a latent state.

## Results

### The phototrophic species Rhodovastum atsumiense G2-11 acquired MGCs from an unknown alphaproteobacterial MTB by recent HGT

**Fig. 1:**
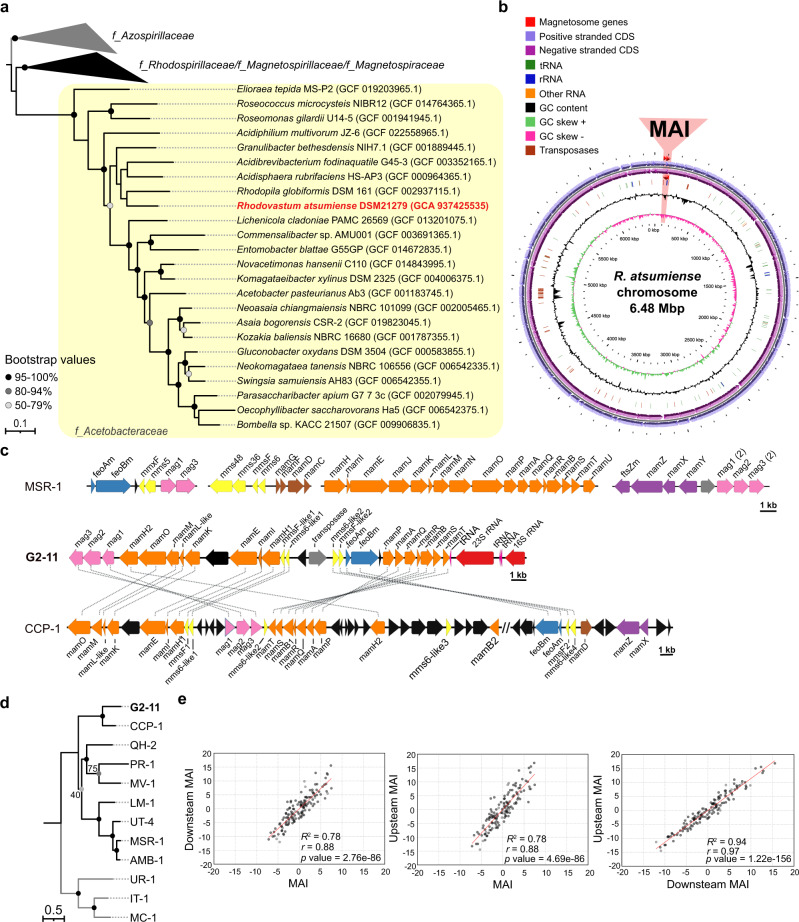
Phylogeny, chromosome, and MGCs organization of G2-11. **a** The maximum likelihood phylogenetic tree based on ribosomal proteins demonstrates the position of G2-11 (highlighted in red) within family *Acetobacteraceae* (highlighted in the yellow box). The *Azospirillaceae* family was used as an outgroup based on the latest *Alphaproteobacteria* phylogeny. Branch length represents the number of base substitutions per site. Values at nodes indicate branch support calculated from 500 replicates using non-parametric bootstrap analysis. Bootstrap values <50% are not shown. The families are labeled according to GTDB taxonomy. **b** Circular map of the G2-11 chromosome. The region of magnetosome genomic island (MAI) is highlighted in red. **c** Organization of the G2-11 MGCs in comparison to MSR-1 and the uncultivated MTB CCP-1. Magnetosome genes are colored according to their position within the magnetosome operons of MSR-1. Connecting dotted lines indicate synteny between the G2-11 genes and CCP-1. **d** Maximum likelihood phylogenetic tree of concatenated amino acid sequences of the shared MamKMOPAQBST proteins for G2-11 and the MTB strains: uncultivated calcium carbonate producing MTB CCP-1, *Magnetospira* sp. QH-2, *Ca.* Terasakiella magnetica PR-1, *Magnetovibrio blakemorei* MV-1, *Ca.* Magneticavibrio boulderlitore LM-1, *Magnetospirillum* sp. UT-4, *M. gryphiswaldense* MSR-1, *M. magneticum* AMB-1. *Ca.* Magnetaquicoccus inordinatus UR-1, *Magnetococcus marinus* MC-1, and *Magnetofaba australis* IT-1 were used as an outgroup. Branch length represents the number of base substitutions per site. The exact values of branch support are indicated at nodes if deviate from 100% (calculated from 500 replicates using non-parametric bootstrap analysis). **e** Distribution of z-normalized tetranucleotide frequencies in the G2-11 MAI region in comparison to the flanking regions (Upstream MAI and Downstream MAI). Each dot represents a tetranucleotide combination. Pearson’s *r*, coefficient of determination *R*^2^, and the *p* values are shown on the graphs.

In a systematic database search for novel MGCs, we identified several orthologs of known magnetosome genes in the recently released draft genome sequence of the culturable anoxygenic phototroph *Rhodovastum atsumiense* G2-11 [25]. This finding was unexpected as, after isolation of G2-11 from a paddy field more than 20 years ago, no magnetosome formation has been reported [26]. Furthermore, no MTB has been identified so far among phototrophs or within the *Acetobacteraceae* family to which G2-11 belongs [26] (Fig. 1a).

Since the available genome version (NZ_VWPK00000000) was highly fragmented (226 contigs), and the magnetosome genes were distributed over several contigs with gaps, we first re-sequenced and assembled the complete genome using long reads generated by Illumina and Nanopore technologies. The resulting genome consists of one chromosome (6.48 Mb) and eight plasmids ranging from 10,690 bp to 220,129 bp in size (Fig. 1b, Supplementary Fig. S1). The putative magnetosome genes localize within a single region (27.5 kb) on the chromosome, compactly organized in four operon-like clusters comprising the following genes: *mag123*, (*mms6-like1*)(*mmsF-like1*)*mamH1IEKLMOH2*, (*mms6-like2*)(*mmsF-like2*), and *feoAmBm-mamPAQRBST* (Fig. 1c). They include all genes thought to be essential for magnetosome biosynthesis (*mamIELMOQB*) [27] and appear to be intact, as no obvious frameshifts or nonsense mutations could be detected. Protein alignments using BLASTP suggested that the closest orthologues of the magnetosome genes from G2-11 are found among alphaproteobacterial MTB (Supplementary Table S1). The phylogenetic analysis of concatenated amino acid sequences of the magnetosome proteins MamKMOPAQBST demonstrated that the sequences from G2-11 reliably cluster with those of a recently discovered uncultivated calcium carbonate producing MTB CCP-1 [28] (Fig. 1d). The low completeness of the CCP-1 genome does not allow us to reliably infer the relationship between G2-11 and CCP-1 using phylogenomic markers, e.g., ribosomal proteins, as we conducted for the *Acetobacteraceae* family (Fig. 1a). Nonetheless, CCP-1 has been shown to belong to *Azospirillaceae* based on the 16 S rRNA tree [28], which occupies an outgroup position related to *Rhodospirillaceae* and *Acetobacteraceae*, according to the latest *Alphaproteobacteria* phylogeny [29]. This incongruence in the phylogenetic positions of these two species and their magnetosome genes is best explained by the HGT of the MGCs in G2-11 from an unknown alphaproteobacterial MTB probably related to *Azospirillaceae*. The additional phylogenetic analyses of the two parts of the MGCs individually (MamKMO and MamPAQBST) did not support different evolutionary histories of these parts, suggesting a single transfer of these clusters from the same organism as the most likely scenario (Supplementary Fig. S2).

Although the magnetosome genes from G2-11 and CCP-1 have a close phylogenetic relationship, the comparative analysis of their MGCs revealed considerable differences in their organization. First, G2-11 lacks several accessory magnetosome genes (*mamX*, *mamZ*, and *mamD*), which were previously shown to be universally present in alphaproteobacterial MTB and, although being not essential, are important for proper magnetic crystal formation in MSR-1 and *M. magneticum* AMB-1 [30, 31]. Their absence in G2-11 could be explained by functional differences in the magnetosome biosynthesis pathways, incomplete horizontal transfer of the MGCs, or a secondary loss of these genes in G2-11. Furthermore, the MGCs of CCP-1 are interspersed by >20 genes with no homology to known magnetosome genes (Fig. 1c). In contrast, the compact MGCs in G2-11 include only a few genes that could not be associated with magnetosome biosynthesis.

Tetranucleotide usage patterns are frequently employed as a complementary tool to group organisms since they bear a reliable phylogenetic signal [32]. Likewise, deviations of tetranucleotide usage in a certain fragment from the flanking genome regions can indicate HGT [21]. Comparison of the z-normalized tetranucleotide frequencies of the MGCs (27.5 kb) with the flanking upstream (117.7 kb) and downstream (79.5 kb) fragments showed a considerably lower correlation between them (Pearson’s *r* = 0.88 with both flanking fragments) than between the flanking fragments themselves (Pearson’s *r* = 0.97, Fig. 1e). This indicates a significant difference in the tetranucleotide composition of the MGCs compared to the flanking genomic regions and supports a foreign origin of the magnetosome genes in G2-11 suggested by the phylogenetic analysis. Besides, the presence of a mobile element (transposase) and position of the MGCs directly downstream of a tRNA gene, a common hotspot for integration of genomic islands [33–35], suggests that the MGCs of G2-11 are indeed located on a genomic island, i.e., represent MAI, like in many other MTB [20, 21]. Unfortunately, the lack of other representatives of the genus *Rhodovastum* makes it impossible to infer whether the MAI was transferred directly to G2-11 or the last common ancestor of the genus. Nonetheless, its compact organization and conspicuous tetranucleotide usage suggest a relatively recent HGT event.

### G2-11 does not form magnetosomes under laboratory conditions

Although magnetosome genes discovered in G2-11 comply with the minimal set required for magnetosome biomineralization in MSR-1 [36], no magnetosomes have been detected in this organism. It might have several explanations: (i) the strain might switch to the magnetotactic lifestyle only under very specific, yet not tested, conditions; (ii) it once was able to synthesize magnetosomes in its natural environment but lost this ability upon subcultivation due to mutations before its characterization; (iii) the strain might naturally not exploit magnetotaxis as its genes might be non-functional or not actively expressed. To clarify which of these explanations is most likely, we first tested whether G2-11 can form magnetosomes under different laboratory conditions. To this end, the strain was cultivated photoheterotrophically, anoxic or microoxic, in a complex medium with potassium lactate and soybean peptone, as commonly used for MSR-1 (FSM) [37], as well as in minimal media with different C-sources previously shown to support growth in G2-11 (glucose, pyruvate, L-glutamine, and ethanol) [26]. All media were supplied with 50 μM ferric citrate to provide sufficient iron for magnetite biomineralization. Since magnetosome biosynthesis is possible only under low oxygen tension, aerobic chemoheterotrophic growth of G2-11 was not tested. The best growth was observed in the complex FSM medium and a minimal medium with glucose or pyruvate, whereas L-glutamine and ethanol supported only weak growth (Supplementary Fig. S3). Irrespective of the growth stage, none of the tested cultures demonstrated magnetic response as measured by a magnetically induced differential light scattering assay (Cmag) [38]. Consistently, micrographs of cells collected from stationary phase cultures did not show any magnetosome-like particles (Supplementary Fig. S3). This confirmed that G2-11 indeed cannot biosynthesize magnetosomes, at least under the conditions available for the laboratory tests. During cultivation, we also noticed that G2-11 cells did not move at any growth stage despite the initial description of this organism as motile using a single polar flagellum [26], and containing several flagellum synthesis operons and other motility-related genes. Moreover, the cells tended to adhere to glass surfaces under all tested conditions and formed a dense clumpy biofilm immersed in a thick extracellular matrix (Supplementary Fig. S3a-ii).

Considering that G2-11 generally lacks magnetosomes and appears to have a stationary lifestyle, which is not consistent with magnetotaxis, we assessed whether the maintenance of MGCs comes at fitness costs for the organism. To this end, we deleted the entire region containing the magnetosome genes (in the following, referred to as the MAI region) using the genetic tools we established for G2-11 in this work (Supplementary Fig. S4a, see Materials and Methods for details). After PCR screening, replica plating test, and genome re-sequencing, two of G2-11 ΔMAI mutants were selected for further analysis (Supplementary Fig. S5). These mutants showed no significant differences in the growth behavior compared to the wildtype (WT) when incubated in minimal media supplied with acetate or pyruvate as a sole carbon source (Supplementary Fig. S4b). This finding suggests that the presence of the magnetosome genes neither provides benefits nor poses any substantial metabolic burden for G2-11, at least under the given experimental conditions.

### RNAseq reveals poor expression levels and antisense transcription in the MGCs of G2-11

**Fig. 2:**
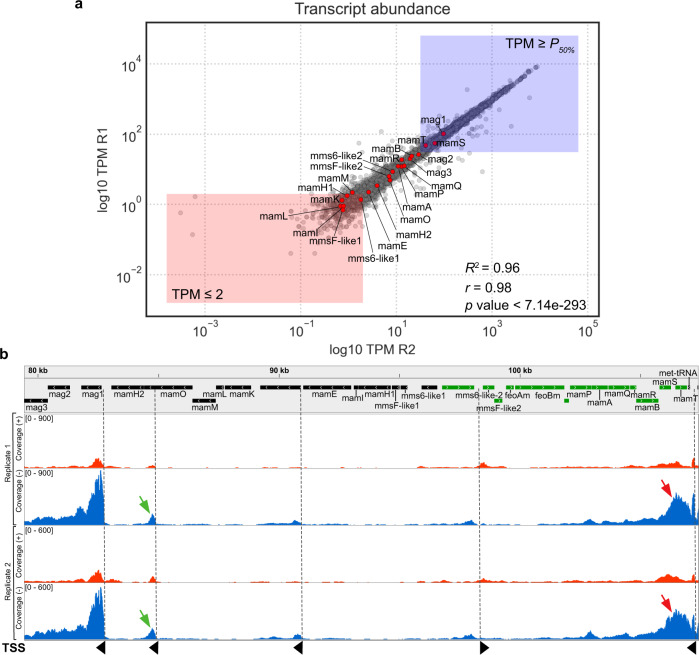
Transcription of the magnetosome genes in G2-11. **a** Log10 of the transcript abundances for all genes in the G2-11 genome presented as TPM (transcripts per million). Red dots represent the magnetosome genes. Red rectangle shows genes with TPM below the threshold, and blue rectangle shows genes with expression levels above median. R1 and R2: biological replicates. Pearson’s *r* and the *p* value is presented on the graph. **b** RNAseq coverage of reads mapped on the positive (red) and negative (blue) strands of the genome in the MAI region. The gray balk shows the gene map: genes encoded on the negative strand are colored in black, on the positive - in green. Red arrows indicate the anti-sense transcription in the *mamPAQRBST* operon. Green arrows indicate the intragenic TSS within *mamO*. TSS are indicated with dashed lines and black arrowheads that show the direction of transcription.

We set on to determine whether the magnetosome genes are transcribed in G2-11. To this end, we analyzed its whole transcriptome for the photoheterotrophic conditions, under which the best growth was observed, in two biological replicates. The expression levels of all the encoded genes calculated as TPM (transcripts per million) demonstrated a high correlation between the two replicates (Pearson’s *r* = 0.98). Most genes of the *(mms6-like1)(mmsF-like1)mamH1IEKLMOH2* cluster were only poorly or not transcribed at all (Fig. 2a, Supplementary dataset). Transcription of *mms6-like1*, *mamF-like1*, *mamL*, *mamH1*, *mamI*, and *mamK*, for example, did not pass the noise background threshold (TPM ≤ 2) in both replicates and were unlikely to be expressed, whereas *mamE*, *mamM, mamH2, feoAm*, and *feoBm* slightly exceeded the threshold in at least one replicate and might be weakly transcribed (Fig. 2a). Although the TPM of *mamO* (TPM = 5.67–6.10, Supplementary dataset) exceeded the background threshold, the coverage plot reveals that the number of mapped reads sharply rises at its 3’-end, whereas the 5’-end has low read coverage (Fig. 2b). This indicates the presence of an internal transcription start site (TSS) and its associated promoter within the coding sequence of *mamO* instead of the full transcription of the gene. Localization of an active promoter within *mamO* was recently described in MSR-1, suggesting that the transcriptional organization of MGCs may be more broadly conserved across MTB than assumed previously [39].

Transcription of genes within the *mag123*, (*mms6-like2*)(*mmsF-like2*), and *mamAPQRBST* clusters significantly exceeded the threshold, with the expression levels of *mag1*, *mamT*, and *mamS* being above the overall median. At the same time, antisense transcription was detected in the *mamAPQRBST* region, with the coverage considerably exceeding the sense transcription (Fig. 2b). This antisense RNA (asRNA) likely originated from a promoter controlling the tRNA gene positioned on the negative strand downstream of *mamT*. Such long asRNAs have the potential to interfere with sense transcripts, thereby significantly decreasing the expression of genes encoded on the opposite strand [40].

In summary, the RNAseq data revealed extremely low or lack of transcription of several genes that are known to be essential for magnetosome biosynthesis (*mamL*, *mamI*, *mamM*, *mamE*, and *mamO*) [27, 41]. Additionally, the detected antisense transcription can potentially attenuate expression of the *mamAPQRBST* cluster that also comprises essential genes, i.e., *mamQ* and *mamB*. Although other factors, like the absence of several accessory genes mentioned above and the potential accumulation of point mutations, might also be involved, the lack or insufficient transcription of the essential magnetosome genes appears to be the primary reason for the absence of magnetosome biosynthesis in G2-11.

### Magnetosome proteins from G2-11 are functional in a model magnetotactic bacterium

Although visual inspection of the G2-11 magnetosome genes did not reveal any frameshifts or other apparent mutations, accumulation of non-obvious functionally deleterious point substitutions in the essential genes could not be excluded. Therefore, we next tested whether at least some of the magnetosome genes from G2-11 still encode functional proteins that can complement isogenic mutants of the model magnetotactic bacterium MSR-1. In addition, we analyzed the intracellular localization of their products in both MSR-1 and G2-11 by fluorescent labeling.

**Fig. 3:**
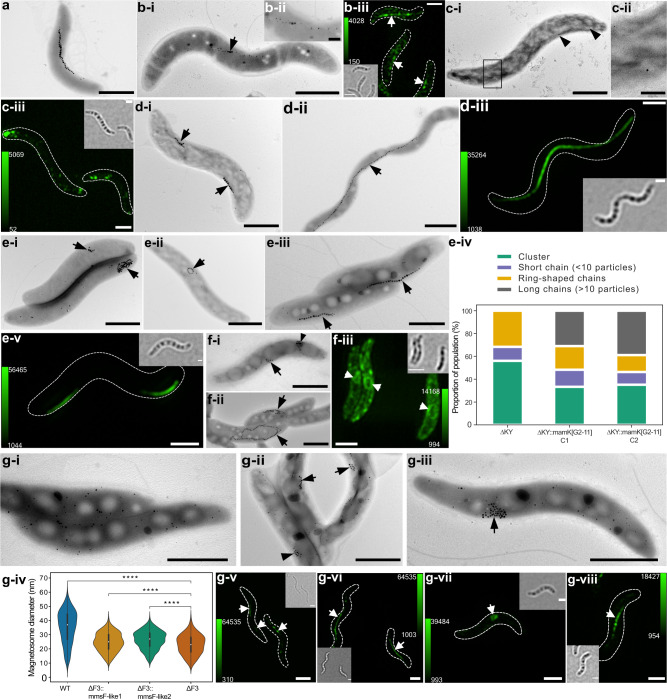
Genetic complementation and intracellular localization of magnetosome proteins from G2-11 in MSR-1 isogenic mutants. **a** TEM micrograph of MSR-1 wildtype (WT). **b** MSR-1 Δ*mamB*::*mamB*_[G2-11]_. **b**-i TEM micrograph and **b**-ii magnetosome chain close-up; **b**-iii) 3D-SIM Z-stack maximum intensity projection of MSR-1 Δ*mamB*::*mamB*_[G2-11]_-mNG. **c** MSR-1Δ*mamQ*::*mamQ*_[G2-11]_. **c**-i TEM micrograph and **c**-ii close-up of the particles; **c**-iii 3D-SIM Z-stack maximum intensity projection. **d** MSR-1 Δ*mamK*::*mamK*_[G2-11]_. **d**-i TEM micrograph of MSR-1 Δ*mamK*; **d**-ii TEM micrograph of MSR-1 Δ*mamK*::*mamK*_[G2-11]_; **d**-iii 3D-SIM Z-stack maximum intensity projection of MSR-1 Δ*mamK*::mNG-_mamK__[G2-11]_. **e** MSR-1 Δ*mamKY*::*mamK*_[G2-11]_. **e**-i-ii Representative cells of MSR-1 Δ*mamKY* mutant showing examples of a short chain, cluster (**e**-i), and ring-shaped chain (**e**-ii); (**e**-iii) TEM micrograph of MSR Δ*mamKY*::*mamK*_[G2-11]_ mutant showing the complemented phenotype; **e**-iv distribution of cells with different phenotypes in the populations of MSR-1 Δ*mamKY* and MSR-1 Δ*mamKY*::*mamK*_[G2-11]_ mutants (*N* > 50 cells for each strain population); **e**-v 3D-SIM Z-stack maximum intensity projection of MSR-1 Δ*mamKY*::mNG-*mamK*_[G2-11]_. **f** MSR-1 Δ*mamJ*::*mamJ-like*_[G2-11]_. **f**-i TEM micrograph of MSR-1 Δ*mamJ*; **f**-ii TEM micrograph of MSR-1 Δ*mamJ*::*mamJ-like*_[G2-11]_; **f**-iii 3D-SIM Z-stack maximum intensity projection of MSR-1 Δ*mamJ*::*mamJ-like*_[G2-11]_-gfp. **g** MSR-1 Δ*F3*::*mmsF-like1*_[G2-11]_ and Δ*F3*::*mmsF-like2*_[G2-11]_. **g**-i TEM micrograph of MSR-1 Δ*F3*; **g**-ii TEM micrograph of MSR-1 Δ*F3*::*mmsF-like1*_[G2-11]_; **g**-iii TEM micrograph of MSR-1 Δ*F3*::*mmsF-like2*_[G2-11]_; **g**-iv magnetosome diameter distribution in MSR-1 Δ*F3* and the mutants complemented with *mmsF-like1*/*mmsF-like2*. Asterisks indicate points of significance calculated using Kruskal–Wallis test (****p < 0.0001); 3D-SIM Z-stack maximum intensity projections of: (g-v) MSR-1 Δ*F3*::mNG-*mmsF-like1*, (**g**-vi) MSR-1 WT::mNG-*mmsF-like1*, (**g**-vii) MSR-1 Δ*F3*::mNG-*mmsF-like2*_[G2-11]_, (**g**-viii) MSR-1 WT::mNG-*mmsF-like2*_[G2-11]_. Scale bars: all TEM micrographs, except close-up images, 1 µm; TEM close-ups, 0.2 µm; 3D-SIM, 1 µm. The calibration bars in 3D-SIM Z-stack projections indicate the minimum and maximum fluorescence intensity. Each 3D-SIM image is supplied with a bright field micrograph of the cells. Black and white arrowheads indicate magnetosomes in TEM and 3D SIM images, respectively.

One of the key proteins for magnetosome biosynthesis in MSR-1 is MamB, as its deletion mutant is severely impaired in magnetosome vesicle formation and is entirely devoid of magnetite crystals [42, 43]. Here, we observed that expression of MamB_[G2-11]_ partially restored magnetosome chain formation in MSR-1 Δ*mamB* (Fig. 3a, b-i, b-ii). Consistently, MamB_[G2-11]_ tagged with mNeonGreen (MamB_[G2-11]_-mNG) was predominantly localized to magnetosome chains in MSR-1, suggesting that the magnetosome vesicle formation was likely restored to the WT levels (Fig. 3b-iii).

Another essential protein MamQ is also involved in magnetosome vesicle formation, and its deletion eliminates magnetosomes in MSR-1 and other magnetospirilla [27, 41]. Expression of MamQ_[G2-11]_ in MSR-1 Δ*mamQ* initiated the biosynthesis of very tiny and scarce magnetosomes (Fig. 3c-i, c-ii). mNG-MamQ_[G2-11]_ was localized in several intracellular patches, which distribution resembled that of the particles observed in the TEM micrographs (Fig. 3c-iii).

MamK is an actin-like filamentous protein, which is an essential structural component of the intracellular “magnetoskeleton” that aligns magnetosomes into linear chains [44]. Deletion of *mamK* in MSR-1 leads to the formation of disrupted short magnetosome chains instead of a continuous long chain typical for the WT (Fig. 3d-i). Expression of MamK_[G2-11]_ in MSR-1 Δ*mamK* resulted in the restoration of a normal magnetosome chain in most of the observed cells (Fig. 3d-ii). mNG-MamK_[G2-11]_ demonstrated linear signal indicating the filament formation [45] (Fig. 3d-iii). Since distinguishing Δ*mamK* from WT or a complemented phenotype can be difficult in shorter cells, we additionally transferred *mamK*_[G2-11]_ into the MSR-1 Δ*mamKY* mutant [46]. In MSR-1 Δ*mamKY*, both magnetosome chains and their positioning are disrupted leading to the formation of magnetosome clusters or very short linear and ring-shaped chains (Fig. 3e-i, e-ii), which represent a more unambiguous phenotype than Δ*mamK*. Complementation of this mutant by a functional *mamK* should result in restoration of long chains, which would be positioned to the outer cellular curvature instead of the geodesic line of the helical cell since *mamY* is absent [46]. Indeed, expression of MamK_[G2-11]_ in MSR-1 Δ*mamKY* resulted in a population that included a considerable number of cells having long (≥10 particles) magnetosome chains, that were absent from MSR-1 Δ*mamKY* (Fig. 3e-iii). Evaluation of >50 cells for each of two randomly selected insertion mutants MSR-1 Δ*mamKY*::*mamK*_[G2-11]_ revealed that the long magnetosome chains were restored in 35-40% of the population (Fig. 3e-iv). Of note, mNG-MamK_[G2-11]_ formed slightly shorter filaments in MSR-1 Δ*mamKY* than in Δ*mamK*, which were also characteristically displaced to the outer cell curvature due to the lack of *mamY* [46] (Fig. 3e-v).

MamJ attaches magnetosomes to the MamK filament in MSR-1, mediating their chain-like arrangement. Elimination of *mamJ* disrupts this linkage, causing magnetosomes to aggregate owing to magnetic interactions [47] (Fig. 3f-i). In MSR-1, MamJ is encoded within the *mamAB* operon, between *mamE* and *mamK*. Within the *(mms6-like1)(mmsF-like1)mamH1IEKLMOH2* cluster of G2-11, there is an open reading frame (ORF) encoding a hypothetical protein that is located in a syntenic locus (Fig. 1c). Although the hypothetical protein from G2-11 and MamJ from MSR-1 differ considerably in length (563 vs. 426 aa), share only a low overall sequence similarity (31%), and are not identified as orthologues by reciprocal blast analyses, multiple sequence alignments revealed a few conserved amino acids at their N- and C-termini (Supplementary Fig. S6). Moreover, in both proteins, these conserved residues are separated by a large region rich in acidic residues (pI 3.3 and 3.2) suggesting that the G2-11 protein might be a distant MamJ homolog. To test if it implements the same function as MamJ, we transferred this gene to MSR-1 Δ*mamJ*. Interestingly, it indeed restored chain-like magnetosome arrangement, which, however, often appeared as closed rings rather than linear chains (Fig. 3f-ii). Despite this difference, it indicated the ability of the hypothetical protein (hereafter referred to as MamJ-like_[G2-11]_) to attach magnetosomes to MamK, suggesting that in the native context, it can have a function identical to MamJ. Consistently, its fluorescently labeled version was often observed in ring-like structures within the cytoplasm of MSR-1 Δ*mamJ*, suggesting that it is indeed localized to magnetosomes (Fig. 3f-iii).

In magnetospirilla, magnetosome proteins MmsF, MamF, and MmxF share an extensive similarity. Their individual and collective elimination gradually reduces the magnetite crystal size and disrupts the chain formation in MSR-1 (Fig. 3g-i; Paulus, manuscript in preparation). The MAI of G2-11 includes two genes, whose products have high similarity to these proteins, designated here as MmsF-like1_[G2-11]_ and MmsF-like2_[G2-11]_. Expression of each of them in the MSR-1 Δ*mmsF*Δ*mamF*Δ*mmxF* triple mutant (Δ*F3*) partially restored the magnetosome size and led to the formation of short magnetosome chains in MSR Δ*F3*::*mmsF-like1*_[G2-11]_ (Fig. 3g-ii) or clusters in MSR-1 Δ*F3*::*mmsF-like2*_[G2-11]_ (Fig. 3g-iii, iv). Consistently, fluorescently tagged mNG-MmsF-like1_[G2-11]_ and mNG-MmsF-like2_[G2-11]_ localized to magnetosomes in the pattern resembling that in the TEM micrographs of the complemented corresponding mutants (Fig. 3g-v, vii), or were perfectly targeted to the magnetosome chains in MSR-1 WT (Fig. 3g-vi, viii).

In G2-11, MamB_[G2-11]_-mNG, mNG-MamQ_[G2-11]_, MamJ-like_[G2-11]_-GFP, mNG-MmsF-like1_[G2-11]_, and mNG-MmsF-like2_[G2-11]_ were patchy-like or evenly distributed in the inner and intracellular membranes (Supplementary Fig. S7). No linear structures that would indicate the formation of aligned magnetosome vesicles were observed in these mutants. As expected, mNG-MamK_[G2-11]_ formed filaments in G2-11 (Supplementary Fig. S7c).

Expression of MamM, MamO, MamE, and MamL failed to complement the corresponding deletion mutants of MSR-1 (not shown). Although detrimental mutations in the genes cannot be excluded, this result can be attributed to the lack of their native, cognate interaction partners, likely due to the large phylogenetic distances between the respective orthologues.

### Transfer of MGCs from MSR-1 endows G2-11 with magnetosome biosynthesis that is rapidly lost upon subcultivation

**Fig. 4:**
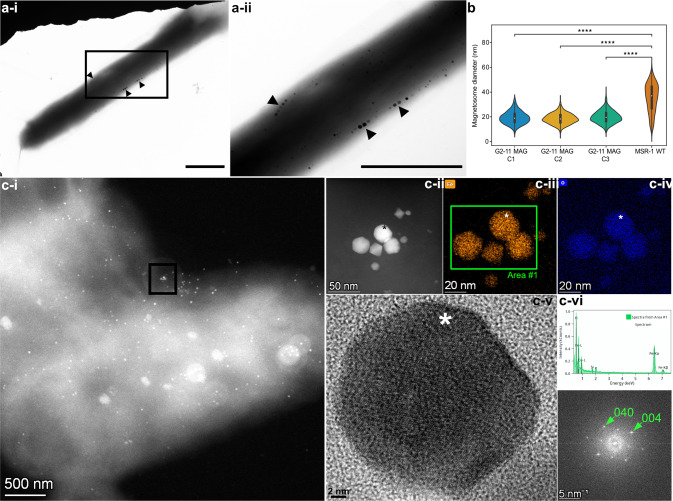
Magnetosome biosynthesis by G2-11 upon transfer of the MGCs from MSR-1. **a** A cell with magnetosomes (i) and a close-up of the area with magnetosome chains (ii). Scale bars: 1 µm. **b** Violin plots displaying magnetosome diameter in three MAG insertion mutants of G2-11 in comparison to MSR-1. Asterisks indicate points of significance calculated using the Kruskal–Wallis test (**** designates *p* < 0.0001). **c** Crystallography analysis of magnetosomes from G2-11 MAG: (c-i) HAADF image of a cell; (c-ii) HAADF image of the cluster from the area shown with a black frame in (c-i); (c-iii) iron (Fe) and (c-iv) oxygen (O) EDS elemental maps of the magnetosome cluster. The peak indicating Cu is an artefact from the copper grid; (c-v) HRTEM image of the magnetosome crystal marked with an asterisk in (cii-iv); (c-vi) EDS spectrum from Area #1 in c-iii; (c-vii) FFT pattern corresponding to the HRTEM in c-v, obtained along the [100] axis of magnetite.

Having demonstrated the functionality of several G2-11 magnetosome genes in the MSR-1 background, we wondered whether, conversely, the G2-11 background is permissive for magnetosome biosynthesis. To this end, we transferred the well-studied MGCs from MSR-1 into G2-11, thereby mimicking an HGT event under laboratory conditions. The magnetosome genes from MSR-1 were previously cloned on a single vector pTpsMAG1 to enable the one-step transfer and random insertion into the genomes of foreign organisms [23]. Three G2-11 mutants with different positions of the integrated magnetosome cassette were incubated under anoxic phototrophic conditions with iron concentrations (50 μM) sufficient for biomineralization in the donor organism MSR-1. The obtained transgenic strains indeed demonstrated a detectable magnetic response (Cmag = 0.38 ± 0.11) [38], and TEM confirmed the presence of numerous electron-dense particles within the cells (Fig. 4), which, however, were significantly smaller than magnetosome crystals of MSR-1 (ranging 18.5 ± 4.3 nm to 19.9 ± 5.0 nm in three G2-11 MAG insertion mutants vs 35.4 ± 11.5 nm in MSR-1 WT, Fig. 4b) and formed only short chains or were scattered throughout the cells (Fig. 4a, c-i). Mapping of the particle elemental compositions with energy-dispersive X-ray spectroscopy (EDS) in STEM mode revealed iron- and oxygen-dominated compositions, suggesting they were iron oxides. High-resolution TEM (HRTEM) images and their FFT (Fast Fourier Transform) patterns were consistent with the structure of magnetite (Fig. 4c). Thus, G2-11 was capable of genuine magnetosome formation after acquisition of the MGCs from MSR-1.

**Fig. 5:**
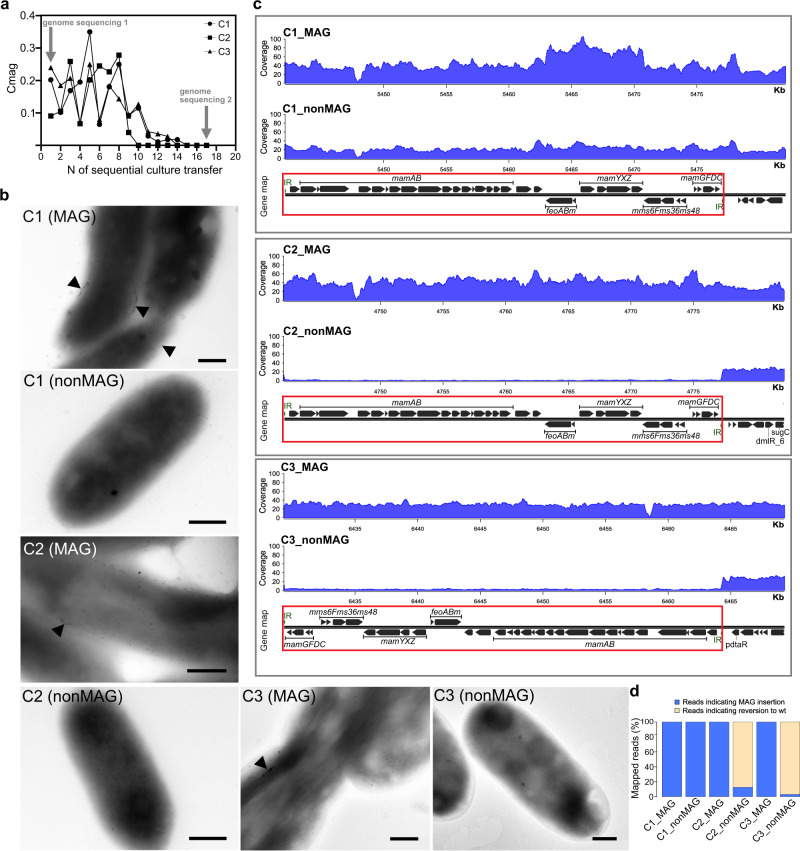
Analysis of the dynamics of magnetosome biosynthesis loss in G2-11 MAG. **a** Change of the magnetic response (Cmag) of three randomLy selected MAG insertion mutants with sequential culture passages. Arrows indicate the timepoints at which the genomes were re-sequenced. **b** TEM micrographs of the cells at timepoint 1 (MAG) and timepoint 17, after the loss of magnetosomes (nonMAG). Scale bars: 0.5 µm. **c** Read coverage normalized to the library size of the MAG and nonMAG mutants. The gene maps show the insertion positions of the MAG cassette (highlighted by red rectangle) within the genome. **d** Bar chart showing the percentage of reads indicating the MAG cassette excision and the cassette presence.

In at least three independent transfer experiments, we noticed that the ability to synthesize heterologous magnetosomes was highly unstable in G2-11 upon subcultivation. The Cmag of the transgenic cultures started to decline soon after the transfer, and the magnetic response became eventually undetectable in all of them after 10–15 daily culture passages. Concurrently, the mutant cells in this non-magnetic state were devoid of magnetosomes. To understand the mechanism of the trait loss, we sequenced the genomes of three randomly selected newly magnetized mutants (hereafter, C1-3) immediately after the genetic transfer, and again after the magnetic response had been lost from the cultures. All three mutants demonstrated a rapid decline in Cmag after the 8th passage (Fig. 5a), whereas the rate at which the cultures transitioned to a completely non-magnetic state (nonMAG) varied among the clones. As expected, TEM observations confirmed the loss of magnetosomes (Fig. 5b). Genome analysis showed that in two out of three insertion mutants (C2 and C3), the entire integrated magnetosome cassette was deleted in their nonMAG descendants (Fig. 5c). Visual inspection of the reads mapped to the insertion locus and the sequences flanking the integrated cassette revealed that a large fraction of the reads (87.4% and 96.9% in C2 and C3, respectively) was mapped to a restored wildtype sequence (except leaving a single nucleotide insertion in place of the deleted cassette), indicating a complete excision of the integrated cassette in most cells (Fig. 5d). Since on pTpsMAG1 the magnetosome cassette is flanked by inverted repeats recognized by the mariner transposase for mobilization and insertion, we believe that these repeats could be recognized and re-used for the excision of the cassette in G2-11, mediated either by intrinsic recombinases or one of many transposases encoded in its genome.

In contrast to C2 and C3, no mutations could be detected in the nonMAG state of C1. This suggests that, in addition to the cassette deletion, other mechanisms to suppress the expression of foreign magnetosome genes, e.g., transcriptional silencing, are likely involved. Besides, the native MGCs present in G2-11 were not affected in either of the mutants. Overall, this experiment demonstrated that although G2-11 can synthesize magnetosomes upon acquisition of the foreign magnetosome genes, their expression imposes a significant negative selection pressure on G2-11, causing the gene deletion (C2 and C3) or potential suppression of expression (in C1).

## Discussion

We present the discovery and comprehensive analysis of dormant MGCs in the non-magnetotactic phototrophic bacterium *R. atsumiense* G2-11. Compared to many well-known silent biosynthetic clusters that control secondary metabolite production pathways [48, 49], this is, to our knowledge, the first evidence not only for the existence of silent genes encoding the biosynthesis of magnetic organelles in any non-MTB, but also prokaryotic organelles in general. Although we cannot completely rule out the possibility that these genes are expressed under yet unknown conditions in the native habitat of G2-11, the early observations of the enrichment culture by Okamura et al. [26] also did not reveal any magnetic behavior. Future studies of this strain in its native environment should help resolve if natural activation of these magnetosome genes can occur. Nonetheless, our results demonstrate that the presence of magnetosome genes does not necessarily correlate with magnetosome biosynthesis, urging researchers to be cautious when interpreting genomic data from public databases in the lack of phenotype or at least the evidence of magnetic enrichment, like in the study of Uzun et al. (2020) [50].

Our analyses suggest a foreign origin of the MGCs in G2-11, deriving them from an unknown alphaproteobacterial MTB likely belonging or related to *Azospirillaceae*. The compact organization, tetranucleotide usage bias and the fact that no MTBs have been revealed among *Acetobacteraceae* to date support a rather recent HGT of the magnetosome genes to G2-11. Despite the lack of other known representatives of the genus *Rhodovastum*, it can be estimated that the HGT event took place at least after the delineation of the contemporary extant *Acetobacteraceae* genera. These data allow us to speculate that HGTs of magnetosome genes might occur more frequently than suggested before [9] and that organisms bearing silent MGCs may contribute to spreading and evolution of magnetosome biosynthesis.

Why were the magnetosome genes not eliminated in G2-11 although not conferring any selective advantage to the host [51]? Several of our findings provide a likely explanation. Since approximately half of the genes within the MGCs of G2-11 are not or only weakly transcribed, and expression of the second half is attenuated by asRNA, we hypothesize that their maintenance within the genome might be neutral to the strain fitness [40]. Indeed, a comparison of the G2-11 ΔMAI mutant and the WT revealed no significant effect of the magnetosome gene presence on the strain growth, at least under the experimental conditions tested, which indicates that the MGCs of G2-11 are likely under neutral selection. Considering the indications that the HGT occurred rather recently, we hypothesize that these genes might not have been eliminated yet due to the short time of their maintenance on the evolutionary scale.

By complementation of isogenic mutants, we demonstrate that magnetosome genes *mamB*, *mamQ*, *mamK*, *mamJ*-like, *mmsF-*like*1*, and *mmsF-*like*2* from G2-11 are functional in MSR-1 and their products localize to magnetosomes. The fact that other genes previously shown to be essential in MSR-1, i.e., *mamM*, *mamO*, *mamE*, *mamI*, and *mamL*, failed to restore the magnetic phenotype in the MSR-1 mutants might be attributed to the protein malfunction in a foreign context, as magnetosome proteins are known to rely on a tight interaction network with other co-evolved magnetosome proteins [42, 52]. Furthermore, the lack of complementation in MSR-1 for some magnetosome genes from distantly related MTB was observed previously [42].

Here, we also tackled the question of whether G2-11 is generally capable of magnetosome biosynthesis if provided with a complete and functional gene set from a foreign donor. The obtained G2-11 mutants were able to produce numerous small magnetosomes, occasionally assembled into short chains. This indicates that G2-11 provides an appropriate and permissive genetic background that supports magnetosome formation upon acquisition of the MGCs from an MTB, although their supposedly suboptimal or imbalanced expression in the foreign host rendered formation of smaller and poorly organized particles. However, unlike previously synthetically magnetized foreign hosts, in which the phenotype was stable [22, 23], G2-11 reproducibly experienced a rapid loss of the magnetic phenotype due to extensive deletions or potential silencing. This finding implies that functional expression of the acquired MGC poses a considerable metabolic burden on G2-11, which stimulates the use of various mechanisms to either avoid unwanted expression or eliminate the genes. These results shed light on the potential state of the G2-11 cells immediately after the HGT of its native magnetosome genes: in the absence of conditions selecting for the magnetosome presence, the phenotype was quickly lost, likely due to the inconsistency between the conferred function and the host’s lifestyle.

In summary, this is the first reported case of a natural occurrence of MGC in a non-magnetotactic, photosynthetic bacterium, which revealed that transcriptional inactivation may serve as an important mechanism for preserving genes encoding a complex trait such as prokaryotic organelle biosynthesis in a latent state within genomes. Future research will show if organisms like G2-11 can also serve as intermediate hosts for further HGT of the silent but functional genes, potentially promoting gene expansion in native communities. Although these MGCs are currently dormant, they may serve for quick adaptation to changing environmental conditions or new niche colonization. Thus, G2-11 provides a rare glimpse into the mostly hidden world of genetic exchange of large gene clusters between microbes.

## Materials and methods

### Strains and conditions

*Rhodovastum atsumiense* strain G2-11 (DSM 21279) and *Magnetospirillum gryphiswaldense* strain MSR-1 (DSM 6361) were routinely cultivated in flask standard medium (FSM, 10 mM HEPES [pH 7.0], 15 mM potassium lactate, 4 mM NaNO_3_, 0.74 mM KH_2_PO_4_, 0.6 mM MgSO_4_ × 7H_2_O, 50 µM ferric citrate, 3 g/liter soy peptone, 0.1 g/liter yeast extract), in 15- or 50-mL screw-capped sterile tubes filled to 3/4 of their volume, at 120 rpm agitation. For phototrophic growth, G2-11 was cultivated in Hungate tubes filled with medium to 2/3 of their volume with the headspace containing 100% N_2_, at a light intensity of 50 µmol/s/m^2^, without agitation. For the test of different conditions for magnetosome formation and the growth comparisons, G2-11 was cultivated in the minimal medium consisting of the mineral base RH2 supplemented with 15 mM of the organic carbon source, 50 µM ferric citrate, and 0.01% yeast extract. Selection for the mutants was carried out on FSM solidified with 1.5% (wt/vol) agar and supplemented with antibiotics: 5 µg/mL of kanamycin (Km) for MSR-1 and G2-11, 6 µg/mL of tetracycline (Tc) for G2-11, 20 µg/mL of Tc for MSR-1, 5 µg/mL of gentamycin (Gm) for G2-11.

*E. coli* WM3064 strains carrying plasmids were cultivated in lysogeny broth (LB) supplemented with 0.1 mM DL-α,ε-diaminopimelic acid (DAP), and 25 µg/mL Km, 12 µg/mL Tc or 15 µg/mL of Gm at 37 °C, with 180 rpm agitation. Characteristics of the strains used in the study are summarized in Supplementary Table S2.

### Genome sequencing and assembly

A closed reference genome of *Rhodovastum atsumiense* strain G2-11T (DSM 21279) was obtained from the genomic DNA extracted from 2–10 mL of stationary cultures with the Zymo Research Midiprep gDNA kit. Sequencing and assembly were performed by mixing Illumina technology and Oxford Nanopore technology. First, for Illumina sequencing, 250 ng DNA was sonicated to a 100–1000 bp size range using the E220 Covaris Focused-Ultrasonicator (Covaris, Inc.). The fragments were end-repaired, then 3′-adenylated, and NEXTflex HT Barcodes (Bio Scientific Corporation) were added using NEBNext DNA modules products (New England Biolabs). After two consecutive cleanups with 1×AMPure XP, the ligated product was amplified by 12 PCR cycles using the Kapa Hifi Hotstart NGS library amplification kit (Kapa Biosystems), followed by purification with 0.6 × AMPure XP. After library profile analysis conducted by an Agilent 2100 Bioanalyzer (Agilent Technologies) and qPCR quantification (MxPro, Agilent Technologies), the library was sequenced on an MiSeq with a MiSeq Reagent Kit v.2 (2 × 250 bp; Illumina Inc.). A total of 3.27 × 106 paired-end reads were obtained. The Illumina reads were trimmed by removing low-quality nucleotides (Q < 20), sequencing adapters, and primer sequences using an internal software based on the FastX package (http://hannonlab.cshl.edu/fastx_toolkit/index.htmL). Reads shorter than 30 nucleotides after trimming were also discarded. For Nanopore sequencing, library preparation was done with 1 μg of the same input DNA following the 1D Native barcoding genomic DNA protocol with EXP-NBD104 and the SQK-LSK109 ligation kit (Oxford Nanopore). The library was sequenced using a Nanopore R9.4.1.revD flow cell (Oxford Nanopore) and the PromethION device with the MinKNOW v.4.0.5 and Guppy v.4.0.11 software. A total of 309 928 reads were obtained with a N50 of 8.07 Kb. Two hybrid assemblies were launched in parallel with Unicycler v.0.4.6 (default options) and Unicycler v.0.4.6 (—sc option for SPAdes) and compared to circularize chromosomes and plasmids [53]. The final assembly resulted in a single chromosome 6.48 Mb in length with a GC content of 69.0% and 8 plasmids (from 220 Kb to 10.69 Kb) with an average GC content of 66.1%. Assembly completeness and contamination were estimated at 99.5 and 0.54%, respectively, using checkM v.1.0.11 [54] with a set of 336 *Rhodospirillales*-specific markers. The automatic annotation was performed with the MicroScope platform (https://mage.genoscope.cns.fr/microscope) [55].

To test the magnetized mutants for the presence or absence of the MAG cassette, genome sequencing was performed again. To this end, the gDNA was extracted as described above. Library preparation and sequencing were performed at Novogene (UK) Co. Ltd using NovaSeq 6000 (Illumina Inc.) with paired-end 2 × 150 bp reads corresponding to 1.0–1.3 Gbp in different samples (estimated coverage 133×). For the raw reads produced with Illumina, adapter trimming and quality control filtering were carried out with *fastp* using standard parameters [56]. Processed reads were mapped to the reference genome using *Bowtie 2* [57] and Geneious (8.1.9) [58]. The read coverage for gDNA re-sequencing in magnetized mutants was calculated using the *bamCoverage* tool, a part of *deepTools* (v. 3.3.2) programs set [59] available on the Galaxy server (https://usegalaxy.eu) [60]. For this, the number of reads that overlap 50 nt bin fragments in the genome was counted and normalized to the number of mapped reads per million. The resulting binned counts per million (CPM) were processed as *bedgraph* or *bigwig* format files. The coverage histograms were visualized using R package *Sushi* v.1.32.0 (Phanstiel, D. H. Sushi: Tools for visualizing genomics data. R package version 1.16.0. https://www.bioconductor.org/packages/release/bioc/htmL/Sushi.htmL (2019)). The fraction of reads indicating reversion to the wildtype locus by excision of the MAG cassette was calculated after mapping reads to the wildtype and the mutant reference genomes by variant calling implemented in Geneious (8.1.9) and checked by visual inspection

### RNA sequencing

RNA sequencing was performed for two biological replicates. For RNA isolation, 200 mL of each replicate culture was grown in 250-mL screw-cap bottles at 28 °C, 180 rpm agitation, with incandescent light (~1500 lux). Cells were harvested at mid-logarithmic phase (optical density at 660 nm [OD660] = 0.130 and 0.159 for replicate 1 and 2, respectively) by centrifugation at 4000 × rpm for 10 min at 4 °C using an Allegra X-15R centrifuge (Beckman Coulter) and flash-frozen in liquid nitrogen before RNA isolation. Total RNA was extracted using hot phenol method [61] with modifications. Briefly, cell pellets were resuspended in 2.5 mL of an ice-cold solution containing 0.3 M sucrose and 0.01 M sodium acetate (pH 4.5). Lysis occurred by careful mixing the resuspended cells with 2.5 mL of hot (65 °C) solution containing 2% SDS and 0.01 M sodium acetate (pH 4.5). The equal volume of hot (65 °C) phenol was added to the lysed cells and mixed by inverting. Tubes were briefly chilled in liquid nitrogen and centrifuged at 4700 rpm for 5 min, 4 °C. The aqueous layer was used for sequential re-extraction with 5 mL of hot (65 °C) phenol, 5 mL of phenol:chloroform:isoamyl alcohol (in proportion 25:24:1, pH 4.5), and chloroform:isoamyl alcohol (24:1). RNA was precipitated by incubation with 2.5 volume of 100% ethanol and 0.1 volume of 3 M sodium acetate (pH 4.5) at −80 °C for 30 min. RNA was pelleted by centrifugation at 4700 rpm for 30 min, 4 °C, washed twice with ice-cold 75% ethanol. After drying in the air, the pellet was resuspended in 200 μL of RNAase-free water. The RNA concentration and quality were controlled by Nanodrop measurements, electrophoresis in 1% agarose gels and the Agilent 2100 Bioanalyzer. Library preparation and RNA sequencing was carried out by Novogene Ltd. (UK). Before library construction, mRNA was enriched using oligo(dT) beads and rRNA was removed using the Ribo-Zero kit. The strain-specific cDNA library was prepared with an Illumina kit according to the manufacturer’s recommendation and sequenced using the NovaSeq 6000. The RNA-seq reads were mapped to the genome using HISAT [62] aligning program with strand-specific parameters. The resulting alignments and the reference annotation were used for *de novo* transcript assembly and prediction of transcription levels with StringTie [63]. The transcription levels were calculated as transcripts per million (TPM) [64]. A threshold of TPM ≥ 2 was applied to define the expressed genes [65].

### Molecular phylogeny

Since the previously built tree based on the 16 S rRNA gene affiliated *Rhodovastum atsumiense* to the phylum *Alphaproteobacteria*, order *Rhodospirillales*, family *Acetobacteraceae* [26], we investigated the evolutionary relationships between strain G2-11 and its *Acetobacteraceae* relatives using whole genome sequences. First the proteome of all non-redundant (1 representative species per genus) closed genomes of *Acetobacteraceae* (LPSN nomenclature, ranked at the order level in GTDB) were downloaded from the public repository RefSeq database at NCBI in April 2022. This database was enriched with the draft genome of the closest relatives of *R. atsumiense*: *Acidisphaera rubrifaciens* strain HS-AP3 and *Rhodopila globiformis* strain DSM 161. The dataset was also completed with a set of genomes from the *Rhodospirillaceae* family and the *Azospirilllaceae* family that was then used as an external outgroup based on the latest *Alphaproteobacteria* phylogeny [29]. A set of 54 proteins composing the 30 S and 50 S ribosome subunits (RPs) were extracted using HMM profiles [66], while the magnetosome proteins sequences were retrieved using the Microscope annotation pipeline. Shared magnetosome proteins MamKMOPAQBST were further used to infer the origin of G2-11 MGCs. For this, genomes of the alphaproteobacterial MTB were used and the closest non-alphaproteobacterial MTB were selected as an external group. The preliminary analysis showed that the currently available metagenome assembled alphaproteobacterial MTB genomes were closely related to those we selected for the phylogenetic reconstruction. Hence, they could not provide any additional information and were not included into the final phylogenetic tree.

For both ribosomal proteins and magnetosome proteins-based trees, amino acids sequences were aligned independently using MAFFT version 7.490 [67] and alignments were trimmed using BMGE [68] setting block length and gap frequency to 3 and 0.5, respectively, and using the BLOSUM30 matrix. Maximum-Likelihood tree was built from the concatenated sequences with IQ-TREE version 2.1.4 [69] and a partition model. For that purpose, a model selection was performed on each protein sequence alignment in the concatenation. Branch support was estimated through non-parametric bootstrap with 500 replicates.

### Molecular and genetic methods

Oligonucleotides applied in this study are listed in Supplementary Table S3. For the complementation experiments, magnetosome genes were PCR amplified from the G2-11 genome using the high-fidelity Q5 polymerase (New England Biolabs, New England USA) and cloned by restriction sites into the pBamII-Tc vector (Supplementary Table S4). To analyze the intracellular localization, the proteins that showed complementation were N- or C-terminally tagged with mNeonGreen (mNG) [70] or GFP that were codon-optimized for MSR-1 and expressed under the control of P_mamDC45_ promoter [71]. The genes were fused to the fluorescent protein gene separated by a GSA linker and cloned into the pBamII-Tc vector using Gibson assembly [72]. For the transfer of magnetosome gene clusters from MSR-1 to G2-11 the previously constructed vector pTpsMAG1 was applied [23].

The plasmids were transferred into MSR-1 and G2-11 by biparental conjugation as described elsewhere [73], with the following modifications for G2-11: the conjugation mixture was inoculated on several selection plates with 10^−2^ to 10^−4^ dilutions, which were incubated for 5–7 days aerobically in the dark, at 28 °C.

### Generation of the ΔMAI mutant of G2-11

To assemble the vectors for deletion of the MAI region in G2-11, the 1.2 kb left (LHR) and right flanking homology regions (RHR) were PCR amplified using specific primers. The LHR was cloned into the vector pAL01-MCS1-Km [36] by *KpnI* and *NotI* restriction sites, the RHR was cloned into pAL02-MCS2-Gm [36] by *KpnI* and *BamHI* (Supplementary Fig. S4). Two versions of Cre-expressing vector pCM157 were tested separately for the region excision: (i) with *cre* gene under the control of inducible *P*_*lac*_ promoter [74], and (ii) under the control of *P*_*nir*_ promoter from MSR-1 [75]. In the culture bearing pCM157-P_*lac*_ expression of Cre was induced by adding 2 mM and 4 mM IPTG, and with pCM157-P_*nir*_ by incubation of the culture under anoxic phototrophic conditions. The deletion protocol was applied as described previously [74] with the following modifications: the cultures were incubated for 6 days under the Cre-expressing conditions with a daily check for the desired mutation by PCR screening. After the band appeared, the culture was plated with dilutions 10^−4^ to 10^−7^. The colonies were scaled-up without antibiotics and checked using replica plating with and without antibiotics for the excision of the integrated plasmids and curing of pCM157. The desired mutation was confirmed by PCR and subsequent genome sequencing using the NovaSeq6000 as described previously.

### Fluorescence 3D-SIM microscopy

For fluorescence microscopy, 2 µL of the cells were immobilized on 1% (wt/vol) agarose pads. 3D-SIM (striped illumination at 3 angles and 5 phases) was performed on an Eclipse Ti2-E N-SIM E fluorescence microscope (Nikon) equipped with a CFI SR Apo TIRF AC 100×H NA1.49 Oil objective lens, a hardware-based perfect focus system (Nikon), LU-N3-SIM laser unit (488/561/640 nm wavelength lasers) (Nikon), and an Orca Flash4.0 LT Plus 17 sCMOS camera (Hamamatsu). 3D-SIM z-series for *R. atsumiense* were acquired at a total thickness of −1.44 to 1.44 μm with 0.12 μm z-step spacing and for MSR-1 at a total thickness of −1.25 to 0.75 μm with 0.12 μm z-step spacing. The exposure time was in the range of 20 to 300 ms at 60% laser power. Fluorescence excitation of mNG was at 403.5 nm and emission was detected at 522.5 nm. Image reconstruction was performed in NIS-Elements 5.01 (Nikon) using the stack reconstruction algorithm with the following parameters. The illumination modulation contrast was set to auto. The high-resolution noise suppression was set to 0.1 [76]. Images were analyzed using ImageJ 1.53c [77].

### Transmission electron microscopy (TEM) and HRTEM

Samples for conventional TEM were concentrated from 2–3 mL cultures by centrifugation, adsorbed on carbon-coated copper grids and imaged using a JEOL-1400 Plus or JEOL-2100 TEM (Japan) at 80 kV acceleration.

High-resolution (HRTEM) images and selected area electron diffraction (SAED) patterns were obtained using the TEM mode of a Talos F200X G2 instrument (Thermo Fisher Scientific, Waltham, MA, USA) at 200 kV accelerating voltage. The same device was used for scanning transmission microscopy (STEM) high-angle annular dark-field (HAADF) images that were collected for both imaging and mapping elemental compositions by coupling the STEM mode with energy-dispersive X-ray spectrometry (EDS).

### Statistical analysis and data visualization

Chromosome and plasmid structures were visualized using CGView 1.7 package [78]. Statistical analysis and graph plotting were carried out using in-house scripts written in Python v. 3.8. Analysis and visualization of the transcription data were conducted using python libraries *pandas 1.0.3* [79] and *matplotlib 3.5.1* [80]. Tetranucleotide-derived z-scores of the MAI and the reference regions were calculated as described in Teeling et al. (2004) [81] using *pyani* (v. 0.2.11) package [82] and a custom script. Correlation was evaluated using the Pearson correlation test.

The magnetosome diameters were measured using imageJ v.1.53c, and the violin plots were constructed applying libraries *seaborn 0.11.2* [83] and *matplotlib*. The Kruskal–Wallis analysis was conducted using the library *statannot* (https://github.com/webermarcolivier/statannot).

## Supplementary information

Table S1

Table S2

Table S3

Table S4

Supplementary Figure S1

Supplementary Figure S2

Supplementary Figure S3

Supplementary Figure S4

Supplementary Figure S5

Supplementary Figure S6

Supplementary Figure S7

Supplementary dataset

## Data Availability

Sequencing reads and the annotated complete genome of strain *Rhodovastum atsumiense* strain G2-11 (DSM 21279) wildtype was deposited to the European Nucleotide Archive database under the BioProject number PRJEB52102. RNA-seq reads and sequencing reads for the genomes of G2-11 mutants generated in this study were deposited to NCBI GenBank database under the BioProject number PRJNA818516.
