# Supplementary material for: Silent gene clusters encode magnetic organelle biosynthesis in a non-magnetotactic phototrophic bacterium
Source: ISME J. 2022 Dec 14;17(3):326–39. doi: 10.1038/s41396-022-01348-y (PMC9938234; doi:10.1038/s41396-022-01348-y)
Supplement: Supplementary file 2 — Table S2 [file 41396_2022_1348_MOESM2_ESM.docx]

**Table S2** Strains used in this study

| **Strain** | **Characteristics** | **Source** |
| --- | --- | --- |
| ***Rhodovastum atsumiense* G2-11** | Wildtype | DSM 21279^1^ |
| ***Rhodovastum atsumiense* ΔMAI** | The region comprising all magnetosome genes, from position 79,446 to 106,923 (27.5 kb) in the chromosome is deleted | This work |
| ***Magnetospirillum gryphiswaldense* MSR-1** | Wildtype, archetype | DSM 6361^2^ |
| ***Magnetospirillum gryphiswaldense* MSR Δ*mamB*** | Δ*mamB* | Awal R.P., manuscript in preparation |
| ***Magnetospirillum gryphiswaldense* MSR Δ*mamM*** | Δ*mamM* | Awal R.P., manuscript in preparation |
| ***Magnetospirillum gryphiswaldense* MSR Δ*mamJ*** | Δ*mamJ* | Lab collection^3^ |
| ***Magnetospirillum gryphiswaldense* MSR Δ*mamK*** | Δ*mamK* | Lab collection^4^ |
| ***Magnetospirillum gryphiswaldense* MSR Δ*mamKY*** | Δ*mamK* Δ*mamY* | Lab collection^5^ |
| ***Magnetospirillum gryphiswaldense* MSR Δ*mamQ*** | Δ*mamQ* | Awal R.P., manuscript in preparation |
| ***Magnetospirillum gryphiswaldense* MSR Δ*mamO*** | Δ*mamO* | Awal R.P., manuscript in preparation |
| ***Magnetospirillum gryphiswaldense* MSR Δ*mamE*** | Δ*mamE* | Awal R.P., manuscript in preparation |
| ***Magnetospirillum gryphiswaldense* MSR Δ*mamI*** | Δ*mamI* | Awal R.P., manuscript in preparation |
| ***Magnetospirillum gryphiswaldense* MSR Δ*mamL*** | Δ*mamL* | Awal R.P., manuscript in preparation |
| ***Magnetospirillum gryphiswaldense* MSR ΔF3** | Δ*mamF* Δ*mmsF* Δ*mmxF* | Uebe R., manuscript in preparation |
| ***E. coli* WM3064** | *thrB1004 pro thi rpsL hsdS* *lacZ*ΔM15 RP4-1360Δ (*araBAD*) 567Δ*dapA1341::[erm pir].* Donor strain for conjugation, auxotroph by DL-α,ε-diaminopimelic acid (DAP) | William Metcalf, UIUC, unpublished |

References

1. Okamura, K., Hisada, T., Kanbe, T. & Hiraishi, A. *Rhodovastum atsumiense* *gen. nov.*, *sp. nov.*, a phototrophic alphaproteobacterium isolated from paddy soil. *J. Gen. Appl. Microbiol.* **55**, 43–50 (2009).

2. Schüler, D. & Köhler, M. The isolation of a new magnetic spirillum. *Zentralbl. Mikrobiol.* **147**, 150–151 (1992).

3. Scheffel, A. & Schüler, D. The acidic repetitive domain of the *Magnetospirillum gryphiswaldense* MamJ protein displays hypervariability but is not required for magnetosome chain assembly. *J. Bacteriol.* **189**, 6437–6446 (2007).

4. Katzmann, E., Scheffel, A., Gruska, M., Plitzko, J. M. & Schüler, D. Loss of the actin-like protein MamK has pleiotropic effects on magnetosome formation and chain assembly in *Magnetospirillum gryphiswaldense*. *Mol. Microbiol.* **77**, 208–224 (2010).

5. Toro-Nahuelpan, M. *et al.* MamY is a membrane-bound protein that aligns magnetosomes and the motility axis of helical magnetotactic bacteria. *Nat. Microbiol.* **4**, 1978–1989 (2019).
