# Supplementary material for: Silent gene clusters encode magnetic organelle biosynthesis in a non-magnetotactic phototrophic bacterium
Source: ISME J. 2022 Dec 14;17(3):326–39. doi: 10.1038/s41396-022-01348-y (PMC9938234; doi:10.1038/s41396-022-01348-y)
Supplement: Supplementary file 5 — Supplementary Figure S1 [file 41396_2022_1348_MOESM5_ESM.pdf]

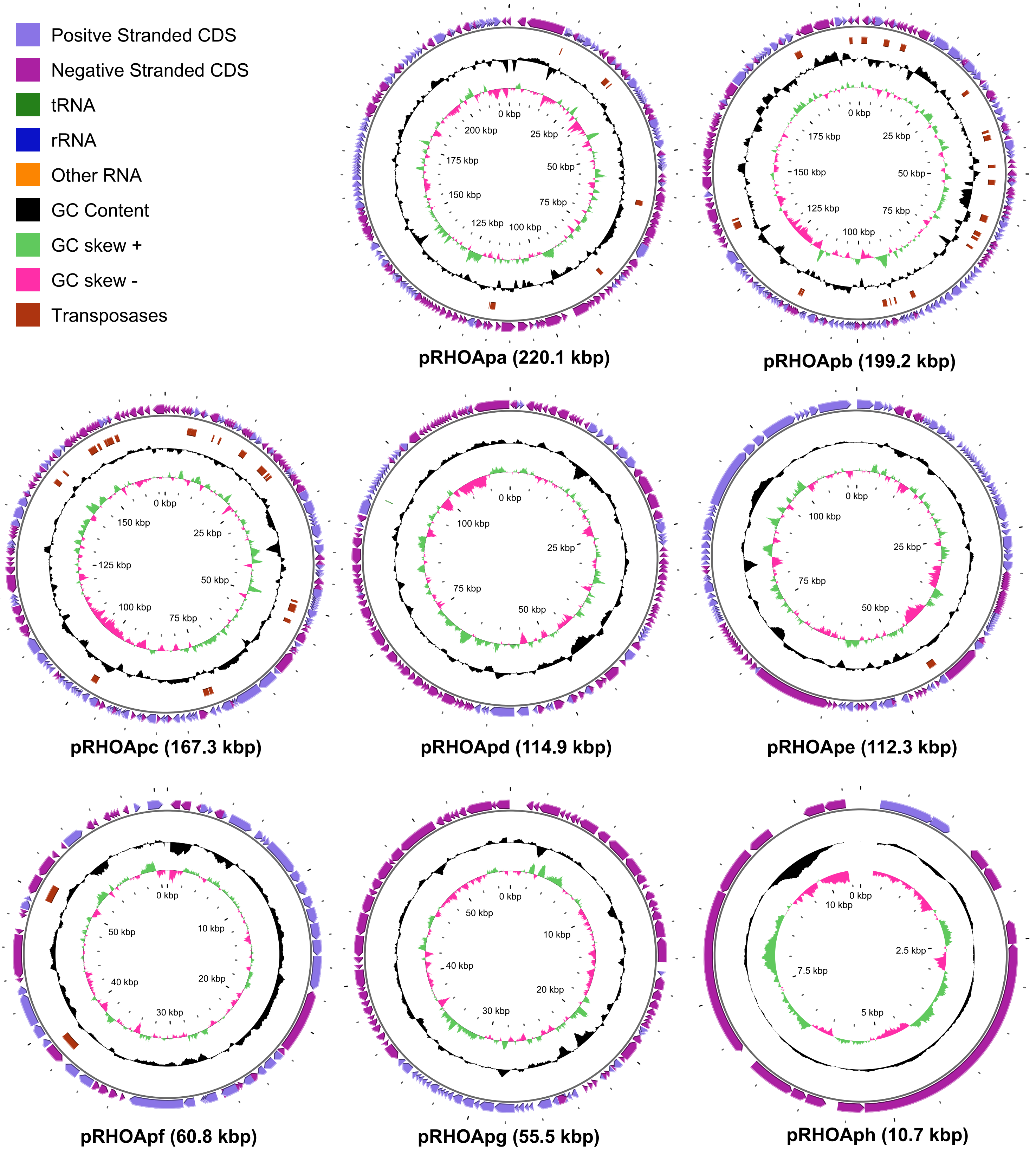

Supplementary Figure S1 Plasmids of *Rhodovastum atsumiense* G2-11
