# Supplementary material for: Silent gene clusters encode magnetic organelle biosynthesis in a non-magnetotactic phototrophic bacterium
Source: ISME J. 2022 Dec 14;17(3):326–39. doi: 10.1038/s41396-022-01348-y (PMC9938234; doi:10.1038/s41396-022-01348-y)
Supplement: Supplementary file 6 — Supplementary Figure S2 [file 41396_2022_1348_MOESM6_ESM.pdf]

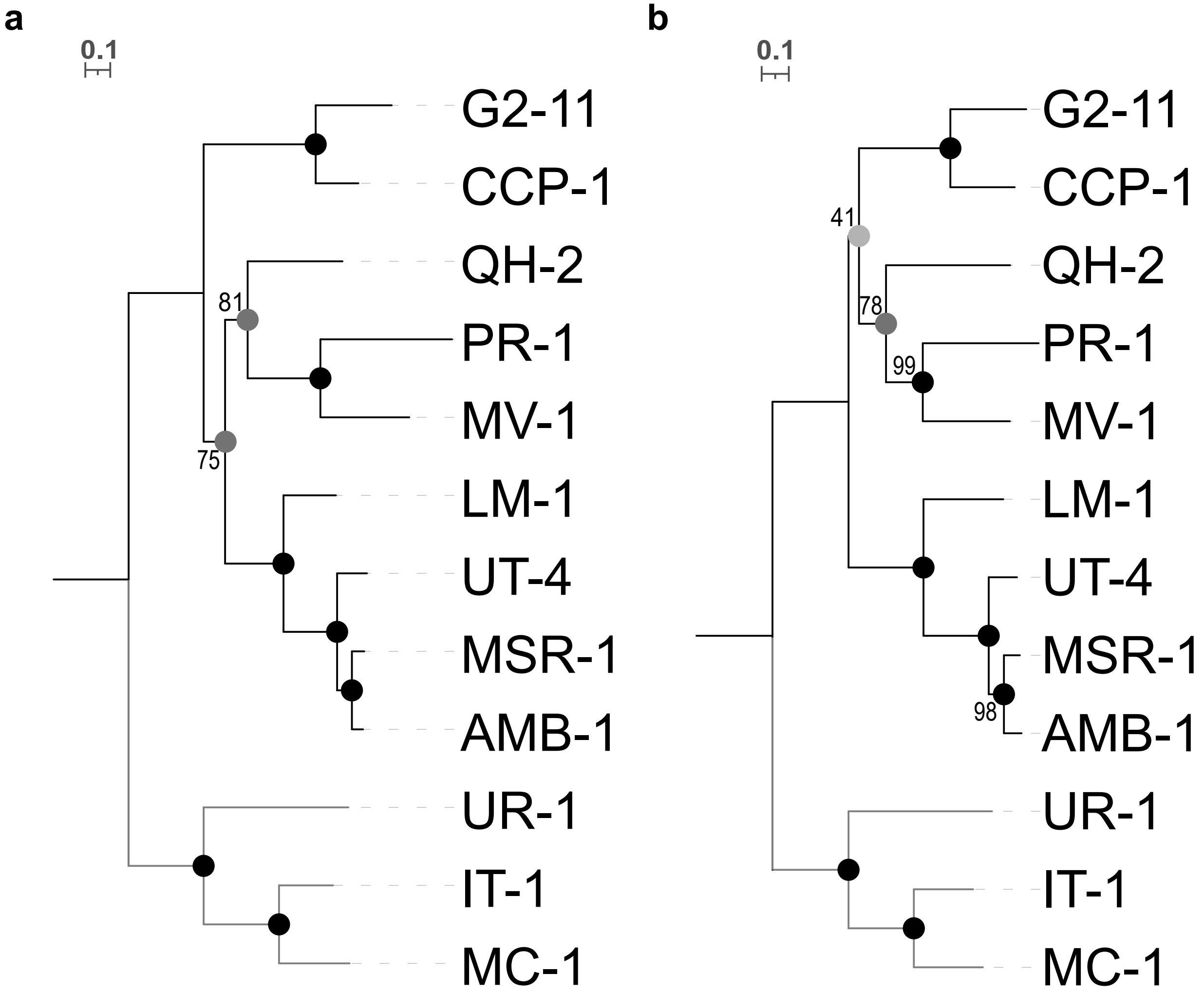

Supplementary Figure S2 Maximum likelihood phylogenetic trees of concatenated amino acid sequences of the MamKMO (a) and PAQBST (b) proteins for G2-11 and the MTB strains: uncultivated calcium carbonate producing MTB CCP-1, *Magnetospira sp.* QH-2, *Ca.* Terasakiella magnetica PR-1, *Magnetovibrio blakemorei* MV-1, *Ca.* Magneticavibrio boulderlitore LM-1, *Magnetospirillum sp.* UT-4, *M. gryphiswaldense* MSR-1, *M. magneticum* AMB-1. *Ca.* Magnetaquicoccus inordinatus UR-1, *Magnetococcus marinus* MC-1, and *Magnetofaba australis* IT-1 were used as an outgroup. Branch length represents the number of base substitutions per site. The exact values of branch support are indicated at nodes if deviate from 100% (calculated from 500 replicates using non-parametric bootstrap analysis). Although MamPAQBST of G2-11 and CCP-1 clusters differently than MamKMO, this positioning is not supported by the bootstraps, suggesting that the same evolutionary history of the two parts cannot be rejected. Besides, the separate position of G2-1/CPP-1 cluster from both the magnetospirilla and QH-2/PR-1/MV-1 groups remains the best supported by the reconstructions with MamKMO.
