# Supplementary material for: Silent gene clusters encode magnetic organelle biosynthesis in a non-magnetotactic phototrophic bacterium
Source: ISME J. 2022 Dec 14;17(3):326–39. doi: 10.1038/s41396-022-01348-y (PMC9938234; doi:10.1038/s41396-022-01348-y)
Supplement: Supplementary file 7 — Supplementary Figure S3 [file 41396_2022_1348_MOESM7_ESM.pdf]

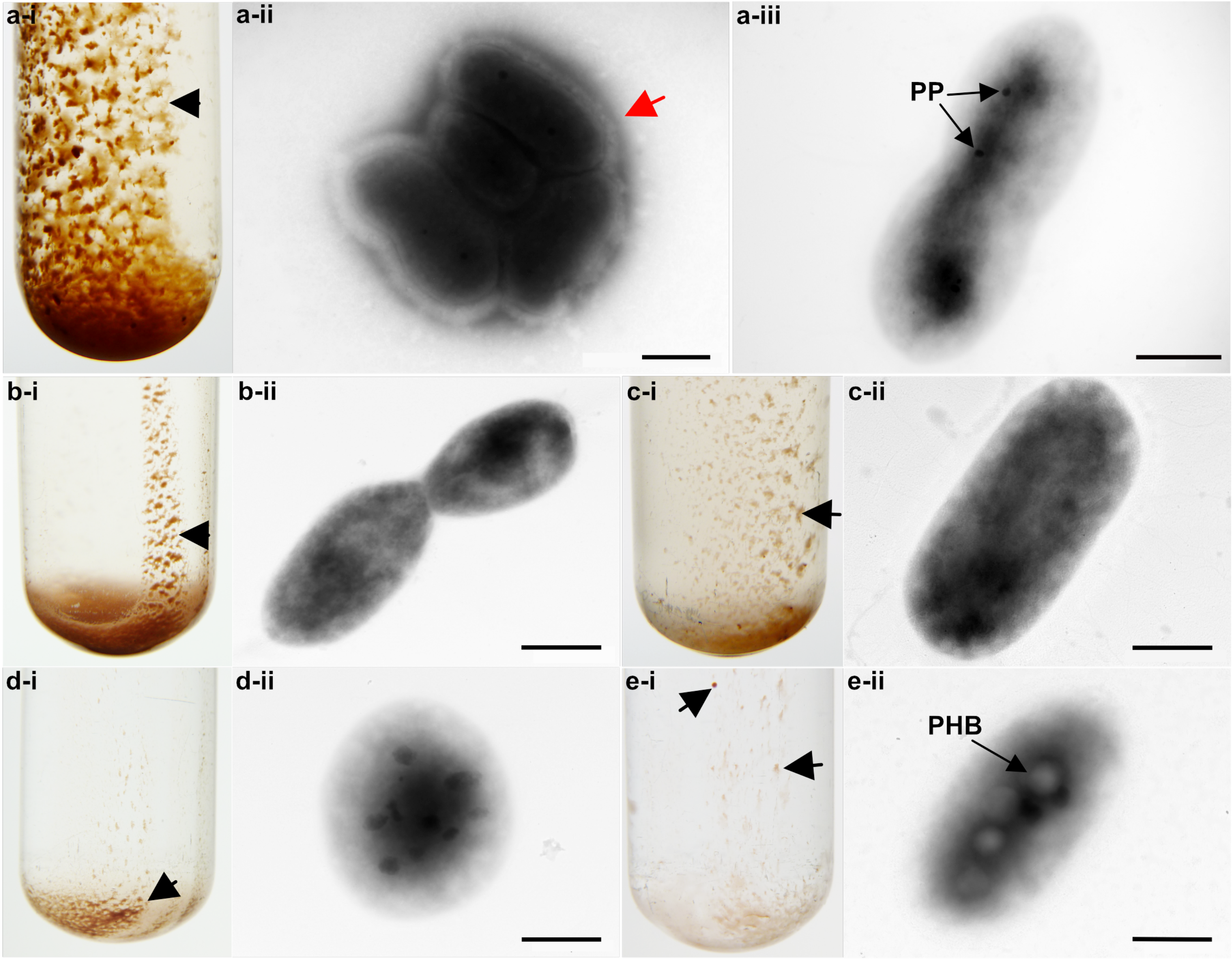

Supplementary Figure S3 Photoheterotrophic cultures (i) and TEM micrographs (ii) of G2-11 cells grown under phototrophic conditions with various carbon sources: (a) complex medium with potassium lactate and peptone (FSM); (b) minimal medium with glucose; (c) minimal medium with pyruvate; (d) minimal medium with L-glutamine; (e) minimal medium with ethanol. Black arrowheads: bacteria adherent to the glass surface; red arrowhead: a matrix encompassing cell clumps; PP: putative polyphosphates; PHB: putative polyhydroxybutyrate inclusions. Scale bars: 1 µm.
