# Supplementary material for: Silent gene clusters encode magnetic organelle biosynthesis in a non-magnetotactic phototrophic bacterium
Source: ISME J. 2022 Dec 14;17(3):326–39. doi: 10.1038/s41396-022-01348-y (PMC9938234; doi:10.1038/s41396-022-01348-y)
Supplement: Supplementary file 8 — Supplementary Figure S4 [file 41396_2022_1348_MOESM8_ESM.pdf]

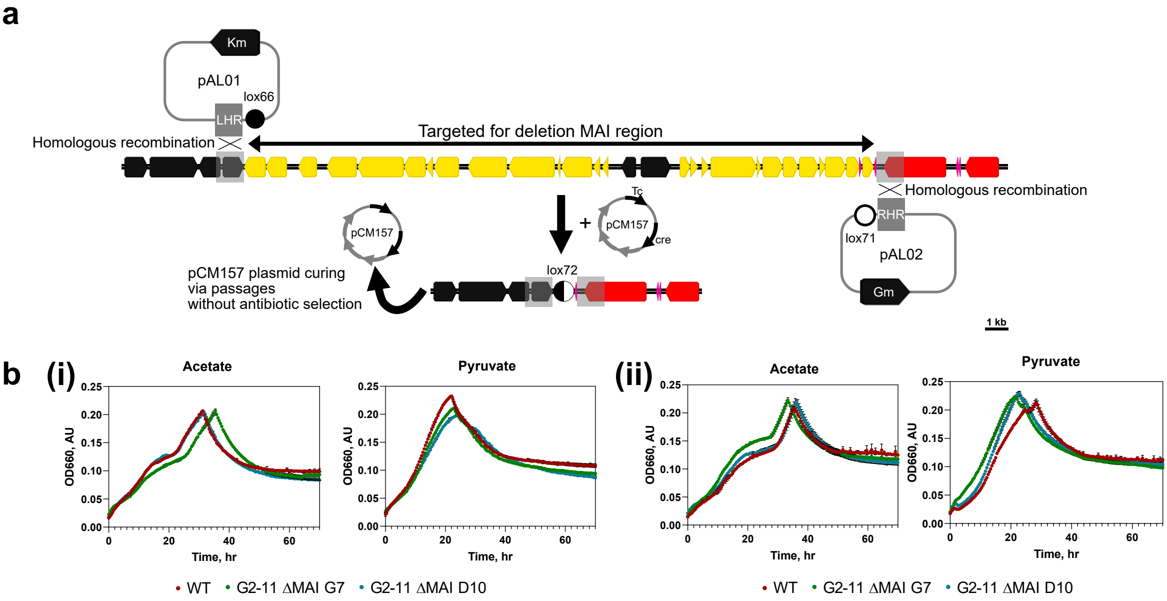

Supplementary Figure S4 Construction scheme and growth analysis of the G2-11 ΔMAI mutants. (a) The MAI region was excised using a Cre-lox-based allele exchange technique with modifications (see 'Materials and Methods' for details). LHR: left homologous region; RHR: right homologous region. (b) Growth curves of two G2-11 ΔMAI clones and the wildtype (WT) cultivated chemoheterotrophically in a minimal medium with acetate or pyruvate as carbon source. Results of two independent experiments are shown (i and ii). Error bars indicate the standard deviation of three biological replicates.
