# Supplementary material for: Silent gene clusters encode magnetic organelle biosynthesis in a non-magnetotactic phototrophic bacterium
Source: ISME J. 2022 Dec 14;17(3):326–39. doi: 10.1038/s41396-022-01348-y (PMC9938234; doi:10.1038/s41396-022-01348-y)
Supplement: Supplementary file 9 — Supplementary Figure S5 [file 41396_2022_1348_MOESM9_ESM.pdf]

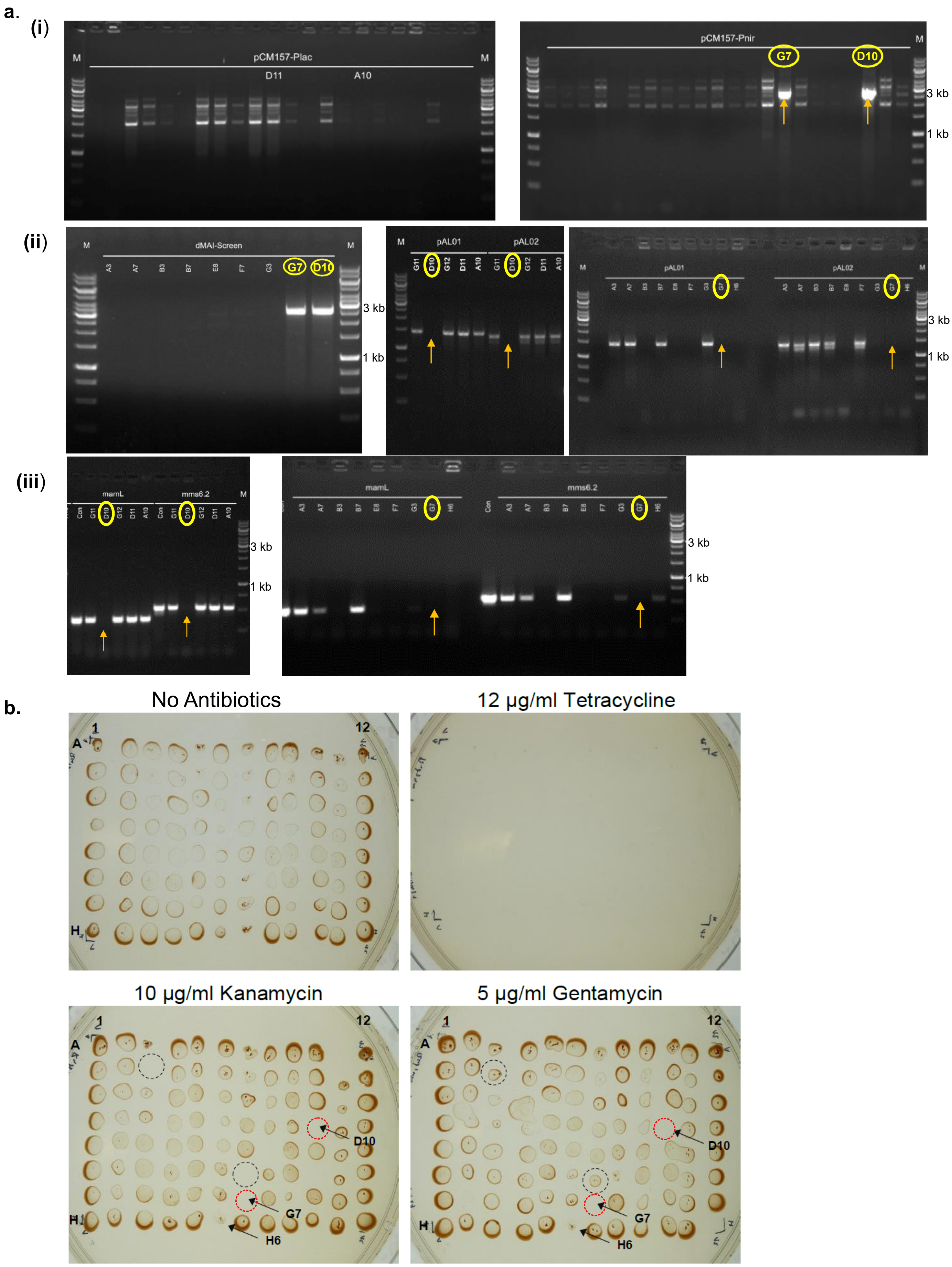

Supplementary Figure S5 Screening for G2-11 ΔMAI mutants. (a) PCR-screening of the transconjugants: (i) screening of the culture for the band indicating deletion in the clones with pCM157-P_{lac} (left) and pCM157-P_{nir} (right). (ii) Screening of the individual clones for (left to right): the band indicating deletion, the excision of the pAL plasmids from the flanking regions. (iii) Test for the presence of the magnetosome genes in the genome of the candidate ΔMAI clones. The clones selected for further work are highlighted by yellow ellipses. (b) Replica-plating of the clones on agar plates with and without antibiotics. The selected mutants are highlighted in red punctuated circles.
