# Supplementary material for: Silent gene clusters encode magnetic organelle biosynthesis in a non-magnetotactic phototrophic bacterium
Source: ISME J. 2022 Dec 14;17(3):326–39. doi: 10.1038/s41396-022-01348-y (PMC9938234; doi:10.1038/s41396-022-01348-y)
Supplement: Supplementary file 10 — Supplementary Figure S6 [file 41396_2022_1348_MOESM10_ESM.pdf]

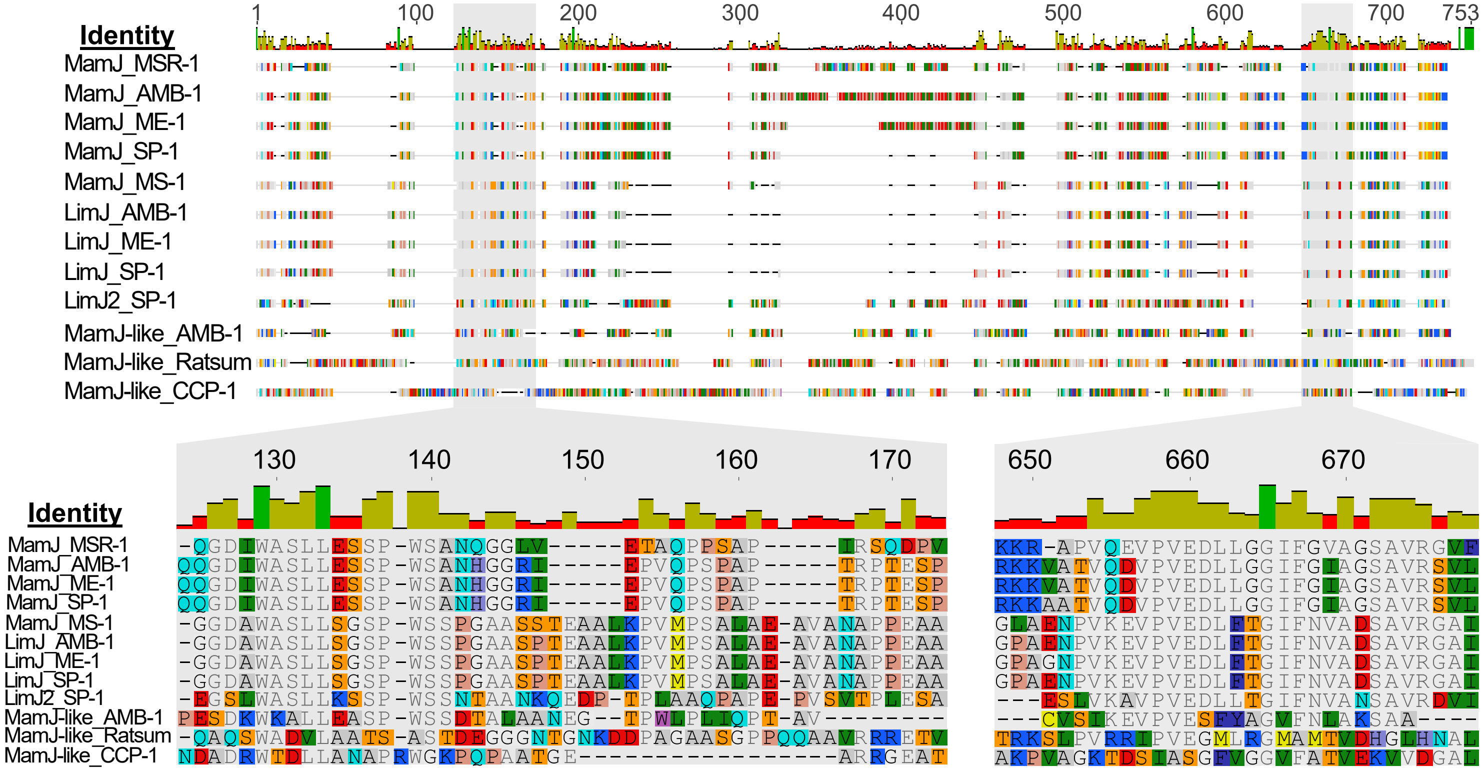

Supplementary Figure S6 Alignment of MamJ-like protein sequences from MTB and G2-11. The fragments that show sequence conservation are shown in close-ups. Note that the gene that occupies the syntenic locus of mamJ in CCP-1 is dissimilar to both MamJ-like from G2-11 and MamJ proteins of magnetospirilla.
