# Supplementary material for: Silent gene clusters encode magnetic organelle biosynthesis in a non-magnetotactic phototrophic bacterium
Source: ISME J. 2022 Dec 14;17(3):326–39. doi: 10.1038/s41396-022-01348-y (PMC9938234; doi:10.1038/s41396-022-01348-y)
Supplement: Supplementary file 11 — Supplementary Figure S7 [file 41396_2022_1348_MOESM11_ESM.pdf]

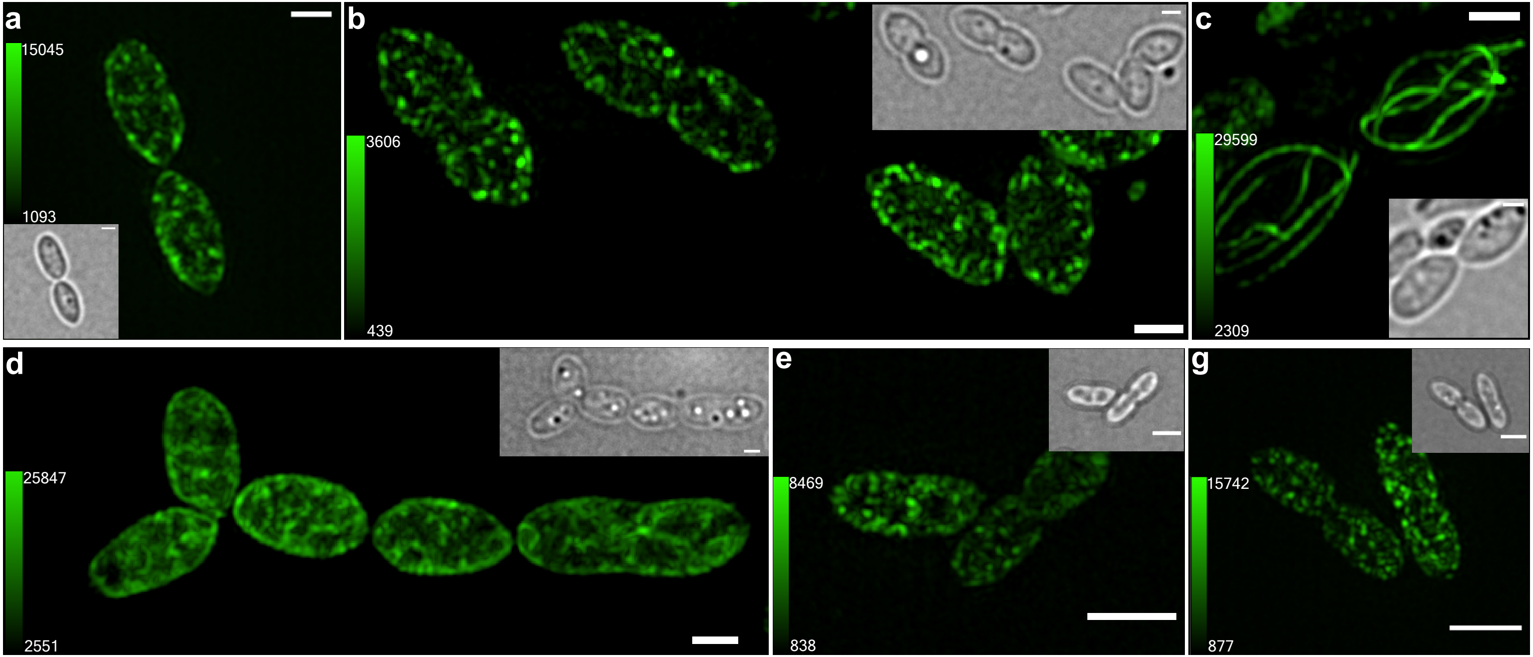

Supplementary Figure S7 3D-SIM Z-stack maximum intensity projection of G2-11 WT expressing magnetosomes proteins tagged by mNeonGreen: (a) WT::mamB[G2-11]-mNG; (b) WT::mNG-mamQ[G2-11]; (c) WT::mNG-mamK[G2-11]; (d) WT::mamJ-like[G2-11]-gfp; (e) WT::mNG-mmsF-like1[G2-11]; (g) WT::mNG-mmsF-like2[G2-11]. Scale bars: (a-d), 1 μm; (e),(g), 2 μm.
